# Impact of Bisphosphonate-related Osteonecrosis of the Jaw on Osteoporotic Patients after Dental Extraction: A Population-Based Cohort Study

**DOI:** 10.1371/journal.pone.0120756

**Published:** 2015-04-16

**Authors:** Yi Fang Huang, Chung Ta Chang, Chih Hsin Muo, Chun Hao Tsai, Yu Fu Shen, Ching Zong Wu

**Affiliations:** 1 Department of General Dentistry, Chang Gung Memorial Hospital, Linkou, Taiwan; 2 School of Dentistry, College of Oral Medicine, Taipei Medical University, Taipei, Taiwan; 3 Department of Emergency Medicine, Far Eastern Memorial Hospital, Taipei, Taiwan; 4 Management Office for Health Data, China Medical University Hospital, Taichung, Taiwan; 5 Department of Orthopedics, China Medical University Hospital, Taichung, Taiwan; 6 Graduate Institute of clinical Medicine, China Medical University, Taichung, Taiwan; 7 Department of Dentistry, Taipei Medical University Hospital, Taipei, Taiwan; 8 Department of Dentistry, Lotung PohAi Hospital, Yilan, Taiwan; Rensselaer Polytechnic Institute, UNITED STATES

## Abstract

**Background and Aims:**

Little is currently known about the risk of developing bisphosphonate-related osteonecrosis of the jaw (BRONJ). This study sought to determine the incidence of BRONJ in osteoporotic patients. We also sought to identify the nature and types of risk factors of osteonecrosis of jaw (ONJ) related to the use of oral bisphosphonates (BPs).

**Materials and Methods:**

Data from the National Health Insurance system of Taiwan. This cohort study included 19,399 adult osteoporosis patients received dental extraction in 2000-2010 (osteoporosis cohort) and 38,669 age and gender matched comparisons selected from dental extraction people without osteoporosis and osteonecrosis history (comparison cohort). All study subjects were followed from the date of their dental extraction (index date) to the development of ONJ and were included in the study up to 2011 or were lost to the study, whichever occurred first. Cox proportional hazard regression was used to estimate the hazard ratio and 95% confidence intervals for the two cohorts.

**Results:**

Patients with osteoporosis had a significantly higher risk to develop ONJ than healthy persons (adjusted HR, 2.05; 95% confidence interval, 1.58–2.65). The risk of ONJ increased with the severity of osteoporosis, no matter whether patient with cancer or not. A cumulative effect of dental extraction frequency may increase the risk of ONJ.

**Conclusions:**

We concluded that ONJ is caused by a number of factors. Osteoporosis and past dental history play the very important roles, while BPs play the synergistic effect.

## Introduction

Many people older than 50 years are suffered from osteoporosis and the incidence of wrist, spine, and hip fractures are expected to increase. Bisphosphonates (BPs) can effectively increase bone density to reduce the incidence of fractures by inhibition of the osteoclast-mediated bone resorption [1]. There are many different routes of BPs administration. Since 2006 the most widely used oral BPs have been alendronate, risendronate, and ibandronate. The most commonly used intramuscular injection treatment involves clodronate, and intravenous therapy has been based on administration of ibandronate, pamindronate, or zoledronic acid. Actually, an estimated 30 million BPs prescriptions are written every year in the United States alone, and more than 190 million prescriptions are written annually worldwide [2].

Bisphosphonate–related osteonecrosis of the jaw (BRONJ) is an adverse drug reaction involving progressive destruction and necrosis in the alveolar bone [3]. The most common sign of BRONJ is bone exposure, and patients usually complain about pain, hypoesthesia, paresthesia, fistula, abscess, swelling, and trismus [4]. In its later stages, BRONJ is usually observed as an infected necrotic jaw, ulcerated, swollen oral mucosa, chronic sinus tracts, facial deformity or impairment of speech, swallowing, and eating. Previous literature indicated if cancer patients received intravenous BPs therapy will increase the occurrence of BRONJ [5], but the risk of developing BRONJ in patients without cancer is still undetermined.

The incidence of BRONJ among BPs recipients has been reported to be 1 case per 100,000 person-years. It is similar to the occurrence of osteonecrosis of the jaw (ONJ) in the general population [6–8]. However, there is no research about investigating the prevalence of ONJ in those who BPs used for the osteoporosis therapy specifically. The previous research by the manufacturer of alendronate, the most widely prescribed oral-BP, pointed out that the incidence of BRONJ is 0.7 cases per 100,000 person-years of exposure in average [9]. The percentage of developing BRONJ after oral BPs administration ranges from 2.5% to 27.3% in another research [10] and most reported cases occurred in patients with malignancies. The estimated cumulative incidence of BRONJ in cancer patients treated with intravenous BPs ranges from 0.8% to 12% [9], and the prevalence of BRONJ in non-cancer patients with osteoporosis may range from 0.02% to 11% [5,11,12]. There is less uniform to compare the percentage of developing BRONJ between the noncancerous osteoporotic patients and those who with malignancies. Therefore, the relationship between oral BPs administration and ONJ is still controversial in those who have osteoporosis. Most patients with osteoporosis diagnosed as ONJ at the time were usually associated with alendronate used. In spite of this, it is difficult to establish the real risk of developing BRONJ for non-cancer patients because of the large number of suspected and unidentified risk factors. In fact, BRONJ can start as a silent and painless bone exposure and in the area difficult to access, so these lesions are hard to be found and felt by most patients. Additionally, the impair life quality during the late stages of BRONJ resembles the side effects from cancer treatments. Clinically the BRONJ is not easily to be diagnosed, hence some authors indicated that under-reporting is noteworthy while claiming that BRONJ is becoming more common [13,14].

There are many proposed risk factors of developing ONJ, such as the duration of BPs exposure, the intravenous BPs administration, periodontal disease, chemotherapy, smoking, glucocorticoid use, and diabetes mellitus. However, the most common immediate facilitating risk factor is the dental trauma, such as dental extraction or previous dento-alveolar surgery, although some spontaneous occurrence cases have also been reported [15–18]. Despite the extensive researches focused on the risk of ONJ in cancer patients with BPs used [19,20], to our knowledge, there is less information about the correlation between ONJ and BPs use in non-cancer patients.

The aims of this population-based cohort study are going to fill the following gaps in knowledge: (1) to investigate the incidence of BRONJ in osteoporotic patients with oral BPs administration, and (2) to estimate the correlation between BRONJ and BPs usage in patients with osteoporosis according to the National Health Insurance system of Taiwan.

## Materials and Methods

### Data source

The Taiwan Bureau of National Health Insurance (TBNHI) set up the National Health Insurance program in March 1, 1995. Approximately 99.5% of residents of Taiwan and 92% of Taiwanese hospitals are enrolled in this program (www.nhi.gov.tw). TBNHI delegated the National Health Research Institutes to contract National Health Insurance Research Databases (HNIRDs) for research proposes. We obtained a copy of the Longitudinal Health Insurance Database 2000, a part of NHIRDs, which included 1 million insurants randomly selected from the original 2000 Registry. This database included all inpatient and outpatient claims and medical care orders from 1996 to 2011. All diseases in the database are identified based on *the International Classification of Diseases*, *9th Revision*, *Clinical Modification* (ICD-9-CM) in all NHIRDs. According to the Personal Information Protection Act, the identification of each insurant was protected by being encrypted by scrambling; this study also approved by an Institutional Review Board at China Medical University Hospital.

### Study subjects

**Fig 1:**
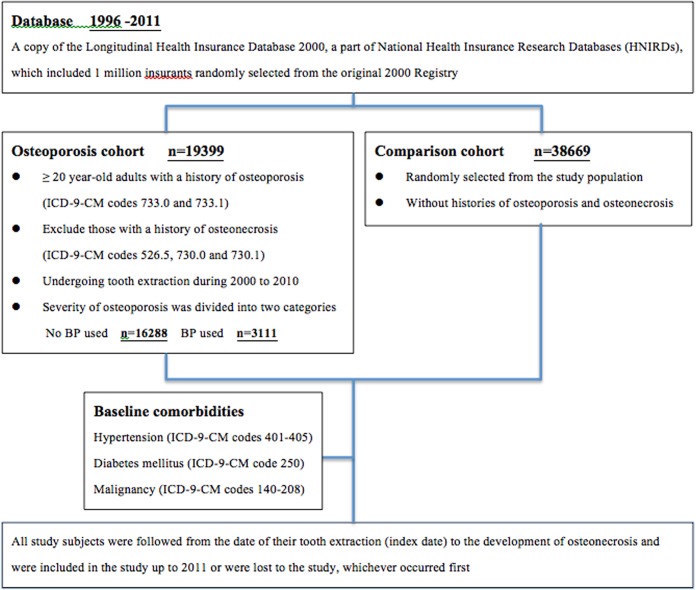
Study flow diagram.

We collected data on adult patients 20 years of age or older who had undergone tooth extraction during 2000 to 2010; these patients formed our study population. In this population, patients with a history of osteoporosis (ICD-9-CM codes 733.0 and 733.1) were defined as the osteoporosis cohort. Those with a history of osteonecrosis (ICD-9-CM codes 526.5, 730.0 and 730.1) were excluded from the study. Those who as comparison cohort had no histories of osteoporosis and osteonecrosis, they were randomly selected from the study population and frequency-matched by age (using a 5-year stratum, for example, ages 20–24, 25–29, and 30–34), gender, and year of tooth extraction. All study subjects were followed from the date of their dental extraction (index date) to the development of ONJ and were included in the study up to 2011 or were lost to the study, whichever occurred first. Baseline comorbidities considered in this study included hypertension (ICD-9-CM codes 401–405), diabetes mellitus (ICD-9-CM code 250), and malignancy (ICD-9-CM codes 140–208). The severity of osteoporosis was divided into two categories, based on the BPs used (Fig 1).

### Statistical analysis

A chi-square test was used to test the difference of demographics and baseline comorbidities between two cohorts. The incidence of ONJ (per 10,000 person-years) was counted in the two cohorts. Cox proportional hazard regression was used to estimate the hazard ratio (HR) and 95% confidence intervals (CIs) for ONJ compared with the comparison cohort. An adjusted model controlled for age, gender, and all comorbidity. Therefore, the age-, gender- and comorbidity-specific risk for ONJ in osteoporosis patients compared with comparisons were assessed. The association between ONJ and the severity of osteoporosis was estimated, and we also assessed the risk by the annual frequency of dental extraction. The severity of osteoporosis was classified into two groups: mild and serious one based on BPs used or not. In order to investigate the impaction of dental extraction frequency on the risk of ONJ, we defined the total numbers of dental extraction divided by follow-up years as the annual frequency of dental extraction; therefore, the annual frequency of dental extraction into three levels: high, as the first quartile; median, as the second quartile; and low, as the third and fourth quartiles. For the future analysis, we assessed the impact of duration or dosage of BPs use on ONJ occurrence, so the level of duration or dosage of BPs use was stratified by median. Kaplan-Meier analysis was used to plot the cumulative incidence for ONJ and log-rank test was used to test the difference between comparison, mild and serious osteoporosis cohort. All analyses were performed with SAS version 9.2 software (SAS Institute, Cary, NC, USA).

## Results

The final study group included 58,069 subjects. 19,399 osteoporosis subjects were assigned to the osteoporosis cohort and 38,669 subjects without osteoporosis were assigned to the comparison cohort. More than half of the osteoporosis patients were elderly, with a mean age of 65.4 years (standard deviation = 11.2), and more women than men were included in the study (83.1% vs. 16.9%, respectively). About 16% of patients with osteoporosis who received BPs were seriously illness. Compared with the patients without osteoporosis, patients with osteoporosis had a higher prevalence of diabetes, hypertension, and cancer (Table 1).

**Table 1:** Demographic data and comorbidity.

	Osteoporosis (n = 19399)		Comparison (n = 38669)		
	Case no.	%	Case no.	%	*P*-value*
Age (years)					0.07
20–44	540	2.78	951	2.46	
45–64	8772	45.2	17544	45.4	
≥ 65	10087	52.0	20174	52.2	
Gender					0.93
Women	16118	83.1	32140	83.1	
Men	3281	16.9	6529	16.9	
Comorbidity					
Diabetes	4313	22.2	7028	18.2	<0.0001
Hypertension	11346	58.5	18553	48.0	<0.0001
Cancer	1013	5.22	1466	3.79	<0.0001
Bisphosphonates					
No	16288	84.0			
Yes	3111	16.0			

*Chi-square test.

During the study, the osteoporosis cohort had a higher incidence of ONJ than the comparison cohort (11.72 vs. 5.32 per 10000 person-years, respectively), with a HR of 2.05 (95% CI = 1.58–2.66) after controlling for age, gender, hypertension, diabetes, and cancer (Table 2). Gender-specific risks were 1.89- and 3.19- folder higher among the osteoporosis cohort than among men and women in the comparison cohort. Age-specific risk was only higher among older subjects, and the difference in risk was statistically significant (HR = 2.95; 95% CI = 2.09–4.17). No matter subjects with comorbidity or not, osteoporosis patients still had a higher risk of ONJ than comparison cohort.

**Table 2:** Incidence and hazard ratio for the osteonecrosis cohort compared to the comparison cohort.

	Osteoporosis		Comparison		HR (95% CI)	
	Case no.	IR^†^	Case no.	IR	Crude	Adjusted^a^
Overall	121	11.72	110	5.32	2.20 (1.70–2.85)**	2.05 (1.58–2.65)**
Age, year						
20–64	40	7.30	56	5.12	1.43 (0.95–2.14)	1.32 (0.88–2.00)
≥ 65	81	16.72	54	5.53	3.02 (2.14–4.26)**	2.95 (2.09–4.17)**
Gender						
Women	98	11.13	96	5.47	2.03 (1.54–270)**	1.89 (1.42–2.51)**
Men	23	15.15	14	4.44	3.42 (1.76–6.64)**	3.19 (1.63–6.24)**
Comorbidity						
No	29	7.47	45	4.41	1.70 (1.06–2.71)*	1.62 (1.01–2.58)*
Yes	92	14.29	65	6.19	2.31 (1.68–3.17)**	2.28 (1.66–3.14)**
Diabetes						
No	84	10.26	80	4.64	2.21 (1.63–3.00)***	2.09 (1.53–2.84)***
Yes	37	17.36	30	8.73	1.99 (1.23–3.22)**	1.95 (1.20–3.16)**
Hypertension						
No	40	8.78	53	4.69	1.88 (1.25–2.83)**	1.74 (1.15–2.63)**
Yes	81	14.05	57	6.07	2.31 (1.65–3.25)***	2.28 (1.62–3.20)***
Cancer						
No	111	11.22	107	5.33	2.10 (1.61–2.74)***	1.95 (1.50–2.55)***
Yes	10	23.51	3	4.90	4.78 (1.32–17.4)*	4.90 (1.35–17.8)*

CI, confidence interval.

^a^Adjusted model, adjusted for age, gender, hypertension, diabetes, and cancer.

^†^IR, incidence ratio, per 10,000 person-years.

*P<0.05;

**P<0.01.

**Fig 2:**
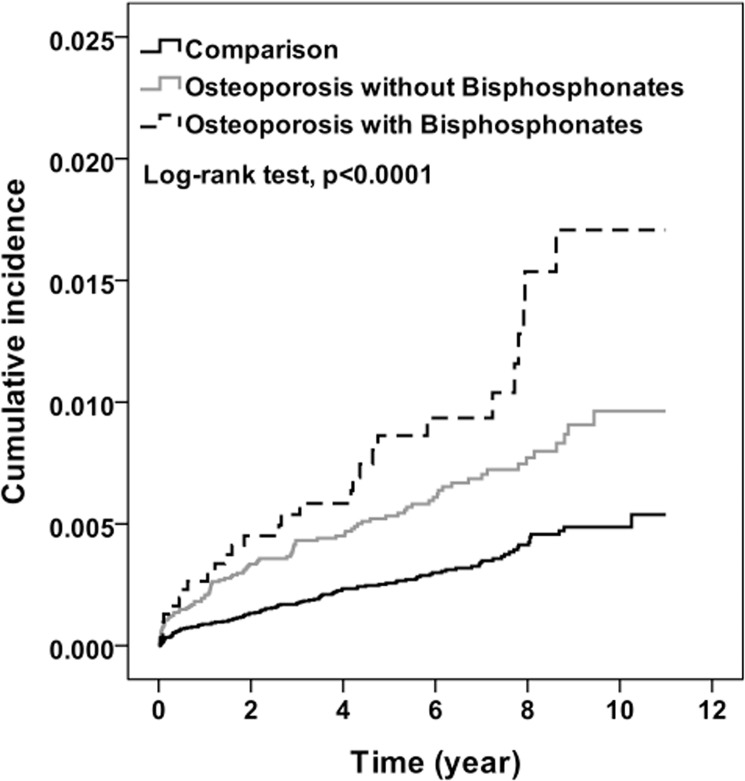
Cumulative incidence for osteonecrosis by osteoporosis and bisphosphonates (Kaplan-Meier analysis).

After 11 years of follow-up, the cumulative incidence of ONJ that increased with the severity of osteoporosis was approximately 0.5% to 1.2% higher among the osteoporosis cohort than among the comparison cohort (Fig 2). Compared with the comparison cohort, osteoporosis patients with serious illness had the highest risk for ONJ, followed by osteoporosis patients with mild disease (HR = 2.71 and 1.91; *P* for trend <0.0001) (Table 3). Patients with severe osteoporosis had had a slightly higher risk than patients with mild osteoporosis, but the difference was not significant. Cancer or non-cancer patients with osteoporosis received BPs had a 1.94- and 1.27-fold risk for ONJ compared with osteoporosis patients without BPs treatment. It did not attach statistical significantly different because of few numbers of ONJ development.

**Table 3:** Incidence and hazard ratio for osteonecrosis among the severity of osteoporosis.

	Case no.	IR^†^	Adjusted^a^ HR (95% CI)	
Overall				
Comparison	110	5.32	1.00	
Osteoporosis				
Mild	93	10.70	1.91 (1.45–2.53)**	1.00
Serious	28	17.17	2.71 (1.77–4.14)***	1.33 (0.86–2.06)
*P* for trend			<0.0001	
Non-cancer				
Comparison	107	5.33	1.00	
Osteoporosis				
Mild	86	10.32	1.84 (1.39–2.45)***	1.00
Serious	25	16.04	2.52 (1.61–3.93)***	1.27 (0.80–2.02)
*P* for trend				
Cancer				
Comparison	3	4.90	1.00	
Osteoporosis				
Mild	7	19.81	1.19 (1.08–16.2)*	1.00
Serious	3	41.72	8.10 (1.62–40.5)*	1.94 (0.49–7.67)
*P* for trend				

HR, hazard ratio; CI, confidence interval.

^a^Adjusted model, adjusted for age, gender, hypertension, diabetes and cancer.

^†^IR, incidence ratio, per 10000 person-years.

**P<0.01;

***P<0.001.

The risk of ONJ was increased with the annual frequency of dental extraction whether in the comparison cohort or mild and severe osteoporosis cohort (*P* for trend <0.0001) (Table 4). The risk of ONJ was also significantly increased with the severity of osteoporosis only among those who received high annual frequency of dental extraction.

**Table 4:** Incidence and hazard ratio for the osteonecrosis cohort compared to the comparison cohort.

Annual Frequency of Dental Extraction	Comparison	Osteoporosis		
		Mild	Serious	*P* for trend
Low*	1.00	1.69 (0.90–3.16)	2.19 (0.75–6.38)	0.08
Median^†^	3.11 (1.81–5.37)***	4.47 (2.41–8.31)***	7.12 (2.87–17.6)***	0.07
High^‡^	15.9 (9.69–26.0)***	39.2 (23.9–64.4)***	46.6 (24.7–87.9)***	<0.0001
*P* for trend	<0.0001	<0.0001	<0.0001	

Adjusted model; model adjusted for age, gender, hypertension, diabetes and cancer.

*Low: < once annually;

^†^Median: once or twice annually;

^‡^High: > twice annually.

***P<0.001.

Table 5 shows the risk of ONJ with different durations or dosages of BPs in the osteoporosis cohort compared with the comparison cohort using Cox proportional hazard model. The risk of ONJ increased significantly with the duration of BPs use, with HR 1.92 in non-use patients and 9.65 in often-use patients, compared to the risk among subjects in the comparisons cohort (trend for test *P*<0.0001). The similar trend of higher risk of ONJ was also found in high dosage group, with HR 1.92 in non-use patients and 7.43 in high dosage patients, compared to the risk among subjects in the comparison cohort (trend for test *P*<0.0001). According to the aforementioned results, we suggest patients with longer durations or higher dosages of BPs use had a significantly higher risk of ONJ than those without BPs treatment.

**Table 5:** Incidence and hazard ratio for osteonecrosis in different duration and dosage of bisphosphonates user.

	Case no.	IR^†^	Adjusted^a^ HR** (95% CI)	
Comparison	110	5.32	1.00	
Bisphosphonates use				
Duration				
None	93	10.7	1.92 (1.45–2.53)***	1.00
Sometime (< 50%)	17	11.58	1.85 (1.10–3.11)*	0.91 (0.54–1.55)
Often (≥ 50%)	11	67.45	9.65 (5.14–18.1)***	4.61 (2.43–8.73)***
*P* for trend			<0.0001	0.0005
Dosage, grams per year				
None	93	10.70	1.92 (1.45–2.53)***	1.00
Low (<1.5)	16	11.49	1.83 (1.08–3.12)*	0.91 (0.53–1.56)
High (≥ 1.5)	12	50.43	7.43 (4.06–13.6)***	3.55 (1.92–6.56)***
*P* for trend			<0.0001	0.009

HR, hazard ratio; CI, confidence interval.

^a^Adjusted model, adjusted for age, gender, hypertension, diabetes and cancer.

^†^ IR, incidence, per 10000 person-years.

*P<0.05;

**P<0.01;

***P<0.001.

## Discussion

Clinically there are different symptoms and signs of ONJ, so it is difficult to make a definite diagnosis. This limitation causes the small numbers of confirmed ONJ cases. Recently, the incidence of BRONJ deriving from retrospective and prospective studies was usually criticized because of the limited sample size and lack of control group. The former report about the association with ONJ and BPs use that was surveyed from medical chart review might easily be overestimated, because they might overlook the poor compliance of osteoporosis patients [21]. Moreover, identifying individual patients with ONJ from the medical records was time-consuming. Although an under- or over-estimation could not be ruled out due to the *ICD-9* diagnosis used in the medical records could be the same as other bone diseases such as osteomyelitis of the jaw. This cohort study reviewed one million documents within the NHIRDs from 2000 to 2010 might be able to overcome the referral bias at the population level. The primary endpoints of our multicenter, retrospectively cohort study were based on examination of the clinical features of ONJ and an assessment of their demographic variables, so the 19,399 osteoporosis patients and 38,669 randomly selected comparison were included in this study.

The dual-energy x-ray absorptiometry scans are used to define the bone mineral density currently, and those who are diagnosed as osteoporosis and can receive BPs therapy under TBNHI support as Z score less than -2.5. Based on the BPs used or not, we distinguished the different severity of osteoporosis in this study. According to NHIRDs, we found that osteoporosis had higher prevalence of illnesses including diabetes, hypertension, and cancer (*P*<0.0001). More than half of the osteoporosis patients were older than 65 years old and 16% of osteoporosis patients were treated with BPs for many years (Table 1). As previous studies mentioned, intravenous BPs therapy for osteoporosis in cancerous patients increases the ONJ occurrence [22], and the incidence of BRONJ ranges from 1% to 10% in these patients [5]. The risk of BRONJ in persons without cancer is still under investigation and there is less information about the association between the severity of osteoporosis and ONJ risk. In order to explore the severity of osteoporosis impact the risk for ONJ, we subdivided the osteoporosis cohort into mild and serious group according to BPs used or not. We hoped to explore the impact of osteoporosis on the ONJ development more precisely after adjusted for age, gender, diabetes, hypertension, and cancer. In this study, the incidence ratio (IR) of ONJ in the osteoporosis patients was 11.72 per 10,000 person-years (Table 2), the data was close to the recent report in Taiwan [23] and within the range of previous research [24]. This result reflected a low prevalence of BRONJ in oral BPs recipients. Although the incidence of ONJ in osteoporosis patients with BPs treatment was slight higher than those without BPs used, there is no significant difference in statistic. According to the result, we also found the IR of ONJ was raised in osteoporosis patients compared to those who without osteoporosis (adjusted HR, 2.05; 95% CI, 1.58–2.65) (Table 3). The risk of ONJ increased with the severity of osteoporosis no matter whether the patients with cancer or not. The above results mean that osteoporosis played major influence on ONJ occurrence (*P*<0.0001). The BPs recipients with osteoporosis have no excessive risk of ONJ than those osteoporosis patients without receiving BPs therapy. This result was also consistent with the previous research which included osteoporosis patients over 50 years in Taiwan [23]. Lin et al. found the risk of ONJ occurrence in osteoporosis patients with alendronate taking is not higher than raloxifene or calcitonin recipients. We speculate that BPs are not the crucial factor to induce ONJ, but BPs might play a synergistic role when ONJ develops. Hence, osteoporosis might play an important role in the ONJ occurrence.

In 2006, Badros et al. [17] indicated that dental extraction is an important initiating factor to develop the ONJ. Before ONJ occurred, 80% of patients had ever received invasive dental treatment such as dental extraction or dental surgery. The clinical guidelines for the cancer patients used BPs recommended strict limitation of oral surgery to reduced the risk of BRONJ [25,26,27]. Our result from NHIRDs indicated that the annual frequency of dental extraction had a significant effect on the ONJ occurrence (Table 4). Both in osteoporosis cohort and comparison cohort, as long as the annual frequency of dental extraction was increased, the risk of ONJ raised substantially. (*P*<0.0001). This finding was not only similar with the previous reports but also coincident to the recent consensus. In 2014, Yuh et al. had represented dental extraction plays major role in the ONJ development [28]. However, the frequency of dental extraction was not estimated in any previous research, they only assessed the invasive dental treatment including dental extraction affects the risk of ONJ. In this study we subdivided both cohorts into low, median and high groups according to annual frequency of dental extraction. We found when the annual frequency of dental extraction is less than twice, there is no significant difference on the ONJ occurrence both in osteoporosis cohort and comparison cohort (*P* = 0.07). Moreover, osteoporosis patients with BPs used had higher risk of ONJ if they received more than twice dental extraction annually (IR, 46.6; HR, 24.7–87.9 *P*<0.0001). The frequency of dental extraction seems to be an important risk factor of ONJ, and we speculated the high frequency could represent the poor oral hygiene care. This result is in agreement with the previous studies, and risk factors of ONJ include poor oral hygiene, a history of dental procedures, and prolonged exposure to high doses of BPs; however, the exact contribution of each risk factor is still ambiguous [29]. In 2005, Marx et al. had pointed out that 84% patients with BRONJ was suffered from periodontitis [30] and many observations both in human and animal models proposed that periodontitis raise the risk of ONJ development [31]. According to our study and previous researches we could infer the close relationship between oral hygiene and ONJ.

In patients at high risk of fractures, it is essential to discover the best way to use BPs, because there is a favorable risk-benefit ratio among these patients. Clinicians need to be aware of the benefits of BPs for reducing the fractures risk significantly, but BPs also brought remarkable morbidity and mortality. It is necessary to weigh the profits of BPs against the risk of BRONJ, so the optimal duration of treatment remains to be defined. There is no information to indicate that stopping oral BPs use will affect the prognosis of any kind of oral surgery, such as dental extraction or decrease the risk of ONJ. Among the different guidelines of BPs administration, Canadian Consensus Practice recommends pondering discontinuation in case of a non-emergent invasive dental procedure in BPs recipients and advises stopping oral BPs between 3 and 6 months before the procedure and starting them again only after healing is achieved. Moreover, some data support a dose-response relationship between ONJ and BPs, but there is little consensus about the relationship between the dosing frequency of BPs used and the ONJ development in the previous research [32]. The correlation of the BRONJ and the dosage or duration of BPs use in osteoporosis patients is still a controversial issue now. Few researches preclude drawing any conclusions about the relationship between these effective treatment characteristics of BPs use and the occurrence of BRONJ. In order to investigate the association with the dosage or duration of BPs use and the ONJ occurrence, we defined the median dosage of BPs leading to ONJ as 1.5g among osteoporosis cohort in this study and used the dosage of 1.5g as baseline to subdivide the BPs recipients into low and high doses of medication groups. According to the time of BPs therapy exceed over half of study period or not, these BPs recipients were divided into often (≥50%) and sometimes (<50%) group (Table 5). Based on our result, compared to those who without osteoporosis, a large cumulative dose of BPs (adjusted HR, 9.65; 95% CI, 5.14–18.1) or long-term use of BPs (adjusted HR, 7.43; 95% CI, 4.06–13.6) raised the risk of BRONJ significantly. Moreover, in the osteoporosis cohort there was significant excess risk for ONJ in the group with longer duration and higher dosage of BPs used. On the other hand, those patients with severe osteoporosis who usually needed high doses or long-term BPs therapy had a marked tendency to develop BRONJ. Our result was similar with the literature mentioned, BRONJ were not a familiar adverse event until a couple of years after BPs used [33]. Previous study suggested ONJ is a rare complication if BPs has been used for less than 5 years [34]. However, the duration and dosage of BPs use may represent the severity of osteoporosis. This finding was coincident to our results (Table 3), there was a significant correlation between the severity of osteoporosis and the probability of ONJ occurrence.

There are several strengths in this study. It provided the preliminary information about the association with the frequency of dental extraction and ONJ occurrence among large and nationally representative population of osteoporosis patients. The large sample size can increase the validity of identifying ONJ, one of the easily miss-diagnosed diseases. The results provided a realistic perspective for impact of BRONJ on osteoporosis patients but there were still some limitations. NHIRDs of Taiwan include both dental and medical records but lack required information for our study such as dental images, periodontal examination (e.g., probing depth, plaque index) and lifestyle behavior (e.g., smoking, betel-nut chewing, alcohol). That caused some difficulties in tracing the reasons of dental extraction and it is hard to further explore the impact of the oral condition or dental status on ONJ development. This provides a further study direction whether the ONJ occurrence is associated with dental condition, smoking or betal-nut chewing habits, and oral hygiene status. In the future prospective studies, it is necessary to explore the possible underlying mechanism of ONJ and wound healing in oral cavity in order to find the effective methods to prevent and treatment BRONJ.

## Conclusion

According to this result, we concluded that the ONJ occurrence is multifactorial. Osteoporosis and past dental history play the major roles in some ways. In contrast, BPs play the synergistic effect. After excluding the impact of cancer factors, the severity of osteoporosis combined the cumulative dosage or the long-term usage of BPs therapy will significantly affect the probability of ONJ occurrence. Appropriate treatment plan for BPs recipients after analyzing every risk factor may avoid the development of ONJ. Good oral hygiene maintenance to reduce the frequency of dental extraction may be one of the best way to prevent ONJ. In addition, it is essential to examine oral cavity for active or anticipated dental issues prior to BPs treatment. A good oral hygiene care is a supreme principle to avoid the BRONJ in osteoporosis patients.
